# Nutritional Evaluation of Turnip Powder in Cereal Blends: A Study on Wheat, Oats, and Turnips

**DOI:** 10.1002/fsn3.70157

**Published:** 2025-05-15

**Authors:** Itrat Fatima Toor, Sanaullah Sajid, Amna Akmal, Zain Ul Abidin, Zeenat Fatima, Suleiman Abdullah Althawab, Lianxian Guo, Tawfiq Alsulami

**Affiliations:** ^1^ Dongguan Key Laboratory of Public Health Laboratory Science, School of Public Health Guangdong Medical University Dongguan China; ^2^ National Institute of Food Science and Technology University of Agriculture Faisalabad Pakistan; ^3^ PMAS‐Arid Agriculture University Rawalpindi Pakistan; ^4^ Department of Food Science and Nutrition, College of Food and Agriculture Sciences King Saud University Riyadh Saudi Arabia; ^5^ The First Dongguan Affiliated Hospital Guangdong Medical University Dongguan China

**Keywords:** food processing, functional foods, nutritional analysis, organoleptic evaluation, turnip powder

## Abstract

This study aimed to evaluate the nutritional enhancement of wheat–oat blends by incorporating turnip powder at varying concentrations (10%, 20%, 30%, 40%, and 50%). The research assessed the impact of these blends on key nutritional components, including moisture, protein, fat, fiber, calcium, phosphorus, iron, and potassium. The wheat‐oat‐turnip blends were prepared and analyzed using standard nutritional methods, including proximate analysis and mineral content measurement. Results showed that increasing the concentration of turnip powder led to significant changes in moisture, protein, fat, fiber, and mineral content (*p* < 0.05). Notably, calcium levels were significantly higher in blends with higher turnip powder concentrations, while other minerals, such as phosphorus and potassium, showed a decreasing trend. Sensory evaluation revealed that a 30% turnip powder blend was most preferred in terms of appearance, aroma, flavor, texture, and overall acceptability. This study demonstrates the potential of incorporating turnip powder into cereal‐based products to improve nutritional profiles while addressing dietary gaps.

## Graphical abstract

This study explores the integration of turnip powder into wheat–oat blends to enhance nutritional profiles and food security. The findings demonstrate that incorporating 30% turnip powder optimizes sensory quality and nutritional benefits, particularly in calcium content, offering a promising approach to improving dietary diversity and promoting healthier eating habits.

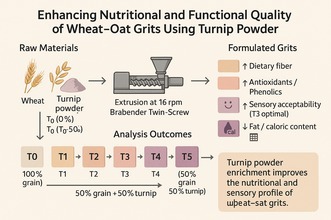

## 1 Introduction

The present‐day world is facing formidable challenges that demand innovative solutions in response to the quest for food security and the promotion of healthier diets. These solutions must not only target nutritional enhancement but also emphasize agricultural sustainability. Among the various strategies being explored, blended food products stand out for their potential to harmonize the nutritional benefits of diverse crops, offering a unique opportunity to effectively address nutritional and environmental needs. This study aimed to evaluate the nutritional enhancement of wheat–oat blends by incorporating turnip powder at varying concentrations and assessed its impact on key nutritional components and sensory characteristics. Research on the development and nutritional evaluation of wheat–oat–turnip blends is emerging as a particularly promising area. Wheat, a staple food in human diets, is a rich source of carbohydrates, dietary fiber, proteins, and various micronutrients essential for a balanced diet. Despite widespread consumption, wheat‐based products often lack certain nutrients and can pose problems for those with gluten sensitivity (Shewry and Hey 2015). In contrast, oats are lauded for their valuable soluble fiber (beta‐glucans), which contributes to heart health, and protein, B vitamins, iron, and antioxidants that combat oxidative stress. Turnips enriched this blend with their high vitamin C, potassium, fiber, and phytochemical contents, offering antioxidant properties and enhancing the nutritional profile of the blend (Warwick 2011; Rasane et al. 2015).

The combination of wheat, oats, and turnips in a single blended product aims to create a more nutritionally balanced offering than any of these components could achieve alone. Such blends not only promise to fill nutritional gaps but also cater to the growing demand for food products that are wholesome, minimally processed, and derived from sustainable sources (Boye et al. 2010; Remans et al. 2011). By combining these ingredients, we leveraged their inherent nutritional strengths and mitigated their limitations, aiming to provide a comprehensive solution to common dietary deficiencies. This tactic contributed to enhanced dietary diversity, serving as a basis for improved food security. Beyond nutrition, blending ingredients often improved the textures, cooking capabilities, and overall sensory appeal of food products. Furthermore, from an environmental standpoint, blends lessen dependence on a single crop and support agricultural biodiversity while offering opportunities to incorporate potentially underutilized crops such as turnips (Mabhaudhi et al. 2016).

The development of these blends involves meticulous formulation to determine the optimal ratios of ingredients, ensuring that the final product delivers targeted nutritional benefits without compromising the flavor or texture. Processing techniques are then applied to transform these raw ingredients into a consumer‐friendly format, a critical step in which innovation meets practicality to ensure that the blends are nutritious and palatable. This stage is particularly challenging, requiring a careful balancing of nutritional integrity with consumer acceptance factors, such as flavor, texture, and aroma. Sensory testing is essential to this process, helping refine the product until it meets the desired standards. The development of wheat–oat–turnip blends has the potential to enhance the nutritional richness, quality, and accessibility of food. These efforts align with the growing consumer demand for wholesome, minimally processed dietary choices that embrace diverse and sustainable ingredients (Simons et al. 2012).

The global trend towards healthier dietary patterns has emphasized the need for functional foods that satisfy hunger and offer significant health benefits. In this context, with their rich nutritional profile, wheat–oat–turnip blends presented an opportunity for innovation in the food industry. This paper presented the development of a wheat–oat–turnip blend aimed at incorporating the health benefits of turnips into a widely consumed food product. This study outlined the rationale behind the blend formulation, its potential health benefits, and its role in promoting healthier eating habits. These ingredients were mixed in pre‐determined ratios to create five distinct experimental groups aimed at evaluating the nutritional impact of varying turnip concentrations. These ratios formed the basis for subsequent product development experiments. The nutritional composition of each ingredient is as follows: Wheat contains 71% carbohydrates, 13% protein, 1.5% fat, 10% fiber, and 4.5% minerals. Oats contain 66% carbohydrates, 17% protein, 7% fat, 11% fiber, and 3% minerals. Turnip powder contains 84% carbohydrates, 1% protein, 0.2% fat, 9% fiber, and 5.8% minerals (Kumar and Singh 2020). Wheat provided essential carbohydrates and proteins, oats contributed soluble fiber and B vitamins, and turnips provided vitamin C and potassium. This combination aimed to provide a balanced diet to address common nutritional deficiencies.

## 2 Materials and Methods

### 2.1 Research Direction

This study aimed to evaluate the nutritional enhancement and sensory qualities of wheat–oat–turnip blends, focusing on the influence of varying turnip powder concentrations on nutritional composition and porridge acceptability.

### 2.2 Selection of Ingredients

Ingredients were selected based on nutritional value, regional sustainability, and availability. The study was conducted at the Food and Nutrition Laboratory of the National Institute of Food Science and Technology, Faculty of Food, Nutrition, and Home Sciences, University of Agriculture, Faisalabad. High‐quality wheat, oats, and fresh turnips ( *Brassica rapa* ) were sourced from the Institute of Horticultural Sciences at the same university, ensuring freshness and traceability. The selection criteria for turnips focused on maturity and size (5–8 cm in diameter), aiming to standardize nutritional content across samples. All reagents used for nutritional analysis were of analytical grade and procured from Sigma‐Aldrich Co., USA, to ensure consistency and reliability in results (Santos et al. 2022).

### 2.3 Preparation of Blends

The preparation process included thorough cleaning of wheat and oats, followed by air‐drying to standardize moisture content at 10%. This prevented mold growth and maintained grain integrity. Turnips were dehydrated at 60°C and pulverized to a fine powder, ensuring uniform distribution in blends. Blend formulations consisted of designated ratios of wheat–oat grits and turnip powder, prepared by boiling with water and simmering. After reaching a creamy consistency and allowing cooling, sugar and skim milk powder were added, and the product was stored in airtight jars. The final product was stored at ambient room temperature (approximately 25°C) within a cool, dark environment. This method was expected to maintain product quality and ensure a shelf life of at least 6 months. The wheat–oat–turnip blends were formulated into six different treatments based on varying concentrations of turnip powder, as shown in Table 1. T_0_ consisted of 100% wheat and oats (1:1) with no turnip addition; T_1_ included 90% wheat and oats (1:1) and 10% turnip; T_2_ was formulated with 80% wheat and oats (1:1) and 20% turnip; T_3_ contained 70% wheat and oats (1:1) with 30% turnip; T_4_ featured 60% wheat and oats (1:1) with 40% turnip; T_5_ was formulated with 50% wheat and oats (Davis and Miller 2017; Lee et al. 2021).

**TABLE 1:** Composition of groups for wheat–oat and turnip blends indicated the percentage ratios across different treatment levels (T_0_ to T_5_).

Treatments	Wheat–oats grits (%) (1:1)	Turnip powder (%)
T_0_	100	0
T_1_	90	10
T_2_	80	20
T_3_	70	30
T_4_	60	40
T_5_	50	50

Abbreviation: T_0_, blend without turnip powder acts as a control.

### 2.4 Proximate Analysis

The nutritional analysis focused on macronutrients, including crude protein, crude fat, dietary fiber, total ash, Nitrogen‐Free Extract (NFE), moisture content, and essential minerals such as calcium, potassium, phosphorus, and iron. Crude protein content was estimated using the Kjeldahl method (Behr Labor, Technik GmbH, Germany) according to AACC method No. 46‐10. Samples (1 g) were digested with sulfuric acid and a digestion mixture containing selenium (Se) and copper (Cu) as catalysts to accelerate the digestion process. The crude protein content was determined using appropriate conversion factors based on the nitrogen content. The conversion factor for wheat was 5.81, while for oats, it was 5.83. Dietary fiber content was quantified using the AOAC method (AOAC 2018). Moisture content was determined using a Memmert 200 hot air oven (Germany) following AACC method No. 44‐15A. The moisture levels were deduced from the pre‐and post‐drying weight differential by drying 10 g samples at 105°C for 24 h (AOAC 2019). The crude fat content was determined using a Soxhlet Apparatus (HTZ 1045 Extraction Unit, Hoganas, Sweden) with n‐hexane (purity ≥ 99%) as the solvent. Furthermore, the nitrogen content was determined using sulfuric acid (H_2_SO_4_, concentrated) as part of the digestion process, combined with a digestion mixture containing selenium (Se) and copper (Cu) as catalysts to facilitate nitrogen conversion into ammonium sulfate (AOAC 2019; Basri et al. 2019).

Ash content was measured using the AOAC Method No. 942‐05. Approximately 4 g of the sample was carbonized on a flame until smoke‐free, then ashed in a muffle furnace (MF‐1/02, PCSIR, Pakistan) at 550°C for 6 h to calculate ash percentage based on residual weight. Nitrogen‐Free Extract (NFE) was calculated to offer insights into the energy‐providing components of food, excluding proteins, fats, fiber, and ash. The formula used was NFE (%) = 100 − (sum of % Moisture, % Crude protein, % crude fat, % crude fiber, and % total ash), highlighting the comprehensive approach to nutritional profiling. Mineral analyses for calcium, potassium, phosphorus, and iron were conducted using wet digestion and specific detection techniques. Phosphorus content was determined spectrophotometrically (Shimadzu UV 240, Japan), potassium through flame emission spectroscopy, and calcium and iron via atomic absorption spectrophotometry. This involved digesting 0.5 g samples with HNO_3_ and HClO_4_, ensuring the precise measurement of mineral concentrations against established standard curves for each mineral (AOAC 2019).

### 2.5 Product Development

Wheat and oat grits were prepared using a method adapted from Bawane and Singhal (2019). The adaptation involved adjusting the extrusion parameters to optimize the grit texture. Specifically, the grits were produced using a Brabender twin‐screw extruder (Brabender GmbH and Co., KG, Duisburg, Germany). After drying, the sample was extruded at 16 rpm to produce fine grits separately. Wheat–oat–turnip blends were then prepared using various formulations, packed in airtight jars, and stored at room temperature (25°C) away from direct sunlight to maintain product integrity. Ready‐to‐use porridge was prepared using all formulations following the modified method of Kayitesi et al. (2010). These blend formulations were combined in 800 mL of water in a 2 L stainless steel saucepan, brought to a boil, and maintained at 100°C for about 5 min. After cooling to 80°C, powdered sugar and skim milk powder were added. The heat was reduced to low, and the mixture was simmered for 30 min, stirring every 5 min to prevent sticking, until the blends softened and the porridge reached a creamy consistency. The product was subsequently stored at room temperature in airtight containers. Microbial analysis (total plate count, yeast/mold) and physicochemical tests (water activity, pH) were conducted on the final product. Samples were analyzed immediately after preparation and at 1, 3, and 6 months of storage at 25°C.

### 2.6 Organoleptic Evaluation

Twenty trained panelists, selected (10 males and 10 females, aged between 20 and 45 years) based on their specific training in food sensory analysis with a general interest in food tasting, evaluated the ready‐to‐use porridge. The evaluation focused on sensory quality characteristics such as appearance, aroma, flavor, texture, mouthfeel, and overall acceptability. A nine‐point hedonic score system was employed, ranging from nine (extremely likely) to one (extremely disliked), as outlined by Meilgaard et al. (2007). To minimize biases, all samples were coded and presented in randomized order. Panelists were briefly oriented on the use of the hedonic scale to ensure consistent scoring. The optimal ratio of wheat, oat, and turnip was determined based on the sensory scores, with statistical analysis performed using ANOVA to identify significant differences among formulations (Miller and Brewer 2018; Brown and Prescott 2022).

### 2.7 Statistical Analysis

The data were analyzed using Analysis of Variance (ANOVA) to assess significant differences among the treatments (T_0_–T_5_) for all measured parameters, including proximate composition and mineral content. Duncan's multiple range test was applied to compare means at a significance level of *p* < 0.05. For sensory evaluation data, ANOVA was used to determine significant differences in sensory attributes (e.g., appearance, aroma, flavor, texture, and overall acceptability). Statistical analyses were conducted using SPSS software version 25 (IBM Corp., USA). All results were presented as means ± standard deviation Teshale et al. (2022).

## 3 Results

### 3.1 Proximate Analysis of Blends

Proximate analysis of the blend prepared using varying concentrations of turnip powder with wheat–oat grits revealed a significant effect of different treatments on moisture, crude protein, crude fiber, ash, and nitrogen‐free extract but a non‐significant effect on crude fat. Moisture was the most important parameter under consideration during the development of dried vegetable products such as blends and flour because it was a direct measure of their shelf life. The moisture content in different turnip powder treatments suggested the product was stable for at least 6 months. The mean values of moisture content ranged from 11.93% to 9.14%, where the highest value was observed in T_0_ (11.93%), followed by T_1_ (11.25%), T_2_ (11.25%), and T_3_ (10.56%), whereas the lowest moisture content was detected in T_5_ (9.14%), followed by T_4_ (9.64%). Crude protein content decreased from T_0_ (11.33%) to T_5_ (8.36%), suggesting that higher turnip proportions in the blend may have diluted the protein content, possibly because of the lower protein content in turnips compared to wheat and oats.

A reduction in crude fat content was observed from T_0_ (3.55%) to T_5_ (2.05%), reflecting the lower fat content in turnips, which affected the overall fat content in the blends as the turnip proportion increased. The crude fiber content decreased slightly from T_0_ (6.37%) to T_5_ (5.25%); while turnips contributed fiber, the overall blend composition affected the total fiber content. The ash content, indicative of the mineral content, decreased from T_0_ (1.94%) to T_5_ (1.06%) and showed a decline in mineral concentration with increased turnip content. NFE content showed a marked decrease from T_0_ (33.94%) to T_5_, with a notable error in T_5_'s reported value, suggesting a decreasing trend in carbohydrates as the turnip ratio increased (Table 2). The pH values ranged from 6.2 to 6.5, indicating slight acidity, while the water activity ranged from 0.65 to 0.68, suggesting good shelf stability and reduced susceptibility to microbial growth. Microbial counts remained below safe thresholds (< 10^3^ CFU/g) during storage.

**TABLE 2:** Proximate composition (%) of wheat–oats and turnip blends across various groups.

Treatments	Moisture	Protein	Fat	Fiber	Ash	NFE
T_0_	11.93 ± 0.25^a^	11.33 ± 0.15^a^	3.55 ± 0.05^a^	6.37 ± 0.10^a^	1.94 ± 0.05^a^	33.94 ± 0.15^a^
T_1_	11.25 ± 0.20^a^	10.89 ± 0.25^b^	3.25 ± 0.05^a^	6.20 ± 0.15^a^	1.88 ± 0.02^a^	33.73 ± 0.18^a^
T_2_	10.75 ± 0.15^b^	10.12 ± 0.20^c^	2.80 ± 0.05^b^	6.15 ± 0.12^a^	1.83 ± 0.03^b^	32.95 ± 0.22^b^
T_3_	10.56 ± 0.10^b^	9.97 ± 0.30^d^	2.50 ± 0.05^c^	6.05 ± 0.10^b^	1.45 ± 0.04^c^	30.75 ± 0.20^c^
T_4_	9.64 ± 0.30^c^	9.50 ± 0.25^e^	2.25 ± 0.05^d^	5.75 ± 0.15^b^	1.22 ± 0.02^c^	28.25 ± 0.15^d^
T_5_	9.14 ± 0.08^c^	8.36 ± 0.23^f^	2.05 ± 0.08^e^	5.25 ± 0.54^c^	1.06 ± 0.12^d^	27.86 ± 0.18^e^

*Note:* Mean ± standard deviation. Values within a column followed by different superscript letters were significantly different (*p* < 0.05) according to Duncan’s multiple range test.

Abbreviations: T_0_, 100% wheat–oats grits (Control); T_1_, wheat–oats grits 90% + turnip powder 10%; T_2_, wheat–oats grits 80% + turnip powder 20%; T_3_, wheat–oats grits 70% + turnip powder 30%; T_4_, wheat–oats grits 60% + turnip powder 40%; T_5_, wheat–oats grits 50% + turnip powder 50%.

### 3.2 Minerals

Estimating the mean square for the mineral composition (calcium, phosphorus, iron, potassium, and magnesium) of blend formulations indicated a significant difference. The mean calcium values demonstrated that the average calcium content ranged from 39.04 to 135.05 mg/100 g, which indicated that T_5_ had the highest calcium level (135.05) and T_0_ had the lowest calcium content (39.04) among all treatments. The mean phosphorus values illustrated the average values from 259.06 to 174.04 mg/100 g, with T_0_ having the highest phosphorus level (259.06) and T_5_ having the lowest phosphorus content (174.04) among other treatments. The mean potassium content was 212.73 mg/100 g, with T_0_ having the highest potassium level (232.06) and T_5_ having the lowest potassium content (212.73). This indicated a dilution effect on potassium levels of increasing turnip content despite turnips being a good potassium source (Table 3).

**TABLE 3:** Comparative analysis of mineral composition (mg/100 g) in wheat–oat and turnip blends, indicating mean values ± standard deviation in different groups.

Treatment	Calcium	Phosphorus	Iron	Potassium
T_0_	39.04 ± 1.54^f^	259.06 ± 0.01^a^	4.35 ± 0.05^a^	259.06 ± 0.13^a^
T_1_	58.03 ± 2.09^e^	242.04 ± 0.06^b^	3.97 ± 0.09^b^	242.04 ± 0.18^b^
T_2_	77.06 ± 0.01^d^	225.04 ± 0.08^c^	3.58 ± 0.04^c^	225.04 ± 0.28^c^
T_3_	96.06 ± 0.11^c^	208.03 ± 0.04^d^	3.16 ± 0.01^d^	221.47 ± 0.45^d^
T_4_	116.02 ± 0.22^b^	191.04 ± 0.32^e^	3.82 ± 0.12^e^	217.14 ± 0.52^e^
T_5_	135.05 ± 0.34^a^	174.04 ± 0.44^f^	2.44 ± 0.38^f^	212.73 ± 0.39^f^

*Note:* Different superscript letters (a–f) within a column indicated significant differences between treatment groups (*p* < 0.05). Means followed by the same letter were not significantly different according to Duncan's Multiple Range Test (DMRT).

### 3.3 Organoleptic Evaluation

The sensory evaluation revealed that 30% turnip powder blends and 70% wheat oat grits were preferred for their appearance, aroma, flavor, texture, mouthfeel, and overall acceptability. This study employed a nine‐point hedonic score system for this evaluation. Product quality depends upon sensory characteristics, and the price is the second factor influencing the product's acceptability.

#### 3.3.1 Appearance

Appearance is the first sensory characteristic perceived by the consumer immediately that predicts consumer acceptance and significantly influences purchasing behavior. Mean values for the appearance score of porridge showed that the appearance of porridge prepared with 30% turnip powder and 70% wheat–oat grits (T_3_) had maximum acceptance to the panel with a score of 8.54, followed by T_2_ and T_1_ having 80% wheat–oat grits and 20% turnip powder (8.24) and T_1_ with 10% turnip powder and 90% wheat–oat grits (7.44). T_3_ scored highest, indicating its visual appeal was most preferred, likely due to a balanced mix, enhancing the porridge's appearance. T_5_ scored lowest, suggesting its appearance may be less appealing, possibly due to ingredient ratios affecting visual attractiveness.

#### 3.3.2 Aroma

T_4_, a 60% wheat–oat grits and 40% turnip powder‐based blend, had the highest aroma score of 7.96, suggesting a more pleasant or appealing aroma, possibly due to a combination of ingredients that released a more favorable scent upon cooking. While T_0_, a 100% wheat–oat grits‐based blend, had the lowest score of 6.45, indicating its aroma may be less inviting or weaker.

#### 3.3.3 Flavor

Playing a significant role in the acceptability of the product is the most important parameter of sensory evaluation. The mean results for the flavor of porridge suggested the full acceptance of T_3_ (7.64), indicating the best flavor balance and palatability, possibly because of an optimal combination of sweetness, bitterness, and other flavor profiles from the blend. In contrast, minimum acceptance was observed for T_5_ (6.35), which could imply flavor imbalance.

#### 3.3.4 Texture

T_3_ again scored highest, indicating a preferred consistency, smoothness, or mouthfeel, which could be due to the physical properties of the blend affecting porridge cohesiveness or creaminess. T_5_'s low score suggested a less desirable texture, possibly grainy or too thick.

#### 3.3.5 Mouthfeel

Like texture, T_3_'s high score reflected a positive reaction to the porridge feel in the mouth, potentially indicating a smooth, creamy, or otherwise pleasant mouthfeel. T_5_'s lower score may denote a less smooth or agreeable mouthfeel.

#### 3.3.6 Overall Acceptability

Porridge prepared with 30% turnip powder and 70% wheat oat grits (T_3_) had maximum acceptance to the panel with a score of 8.54. This suggested that its combination of sensory attributes aligned well with consumer expectations and preferences, followed by T_2_ and T_1_ having 80% wheat–oat grits and 20% turnip powder (8.24) and T_1_ with 10% turnip powder and 90% wheat–oat grits (7.44). T_5_, with the lowest score, indicated its sensory profile may not meet consumer standards for porridge, affecting its acceptability (Table 4).

**TABLE 4:** Mean comparison of the sensory attributes of ready‐to‐use porridge.

Treatments	Appearance	Aroma	Flavor	Texture	Mouthfeel	Overall acceptability
T_0_	7.19 ± 0.11^d^	6.45 ± 0.07^f^	7.12 ± 0.08^c^	7.64 ± 0.02^c^	7.67 ± 0.06^c^	8.13 ± 0.03^c^
T_1_	7.44 ± 0.14^c^	6.55 ± 0.14^e^	7.13 ± 0.07^c^	7.77 ± 0.07^c^	7.74 ± 0.13^c^	8.27 ± 0.04^c^
T_2_	8.24 ± 0.18^b^	7.12 ± 0.12^d^	7.64 ± 0.104^b^	8.00 ± 0.05^b^	8.12 ± 0.11^b^	8.45 ± 0.02^b^
T_3_	8.54 ± 0.24^a^	7.76 ± 0.38^c^	7.64 ± 0.23^b^	8.13 ± 0.11^a^	8.45 ± 0.17^a^	8.65 ± 0.09^a^
T_4_	6.35 ± 0.08^e^	7.96 ± 0.18^a^	6.98 ± 0.11^d^	6.53 ± 0.09^d^	7.37 ± 0.04^d^	7.82 ± 0.04^d^
T_5_	6.15 ± 0.05^f^	6.86 ± 0.11^d^	6.35 ± 0.18^e^	6.17 ± 0.25^e^	7.02 ± 0.09^e^	7.08 ± 0.01^e^

*Note:* Values within a column followed by different superscript letters were significantly different (*p* < 0.05) according to Duncan’s multiple range test.

## 4 Discussion

This study embarked on a detailed exploration aimed at integrating the health benefits of turnips into a widely accepted food product, highlighting a global shift towards healthier eating habits. Specifically, it sought to evaluate how varying concentrations of turnip powder influenced the nutritional profile of wheat–oat blends, with a keen eye on moisture, protein, fat, and fiber content. This investigation aspired to contribute to filling nutritional gaps, promoting dietary diversity, ensuring food security, and advancing environmental sustainability.

The proximate analysis using standard AOAC methods illuminated a trade‐off in nutritional components as the concentration of turnip powder increased. Notably, there was a decrease in moisture, protein, fat, and fiber content, juxtaposed with a significant enhancement in calcium content. This trade‐off highlighted the nutritional recalibration inherent in blending diverse food sources. The reduction in protein content, consistent across the treatments from T_0_ to T_5_, aligned with the anticipated dilution effect, given the lower protein content in turnips compared to wheat and oats. Conversely, the enrichment of calcium in blends with higher turnip concentrations underscored the potential of turnips to fortify food products with essential minerals, aligning with dietary recommendations for calcium intake.

A statistical analysis was performed using ANOVA to identify significant differences between treatments. Moisture content, ranging from 11.93% to 9.14% across treatments, indicated a decreasing trend as the proportion of turnip powder increased. The lowest moisture content in T_5_ suggested that higher turnip powder concentrations may contribute to a drier product, which can be advantageous for shelf stability. Moisture is a critical parameter for dried vegetable products since it directly correlates with shelf life; lower moisture content indicates longer shelf stability, potentially extending the product's usability beyond 6 months.

The decrease in crude fat content with increased turnip powder was consistent with the known lower fat content of turnips. This aspect might be beneficial for consumers seeking lower‐fat dietary options. The decrease in protein and fat content aligned with previous findings, emphasizing the lower protein and fat levels in turnips compared to grains. This research builds upon the foundational work by Santos et al. (2022), who documented the dietary fiber benefits of vegetable incorporation into cereal products, offering a comparative analysis that underscored the novelty and contribution of our findings to the existing body of knowledge. The formulation percentages of wheat, oats, and turnip powder in the blends were predetermined by the researchers, creating intentional variations that ranged from 100% wheat–oats (T_0_) to a 50% wheat–oats and 50% turnip powder composition (T_5_). These formulations were designed to examine the effects of different ingredient ratios on the nutritional profile and sensory qualities of the blends. These proportions were selected based on previous research, which has found that vegetable powder incorporation in cereal blends typically ranges from 10% to 50%, depending on the desired nutritional enhancement and sensory characteristics. Santos et al. (2022) utilized similar turnip concentrations to enhance the mineral content in cereal‐based products, aligning with the current study's goal of optimizing nutritional composition while maintaining sensory acceptance.

The slight decrease in crude fiber content from T_0_ to T_5_ suggested that, although turnips were a source of fiber, the overall fiber content was influenced by the proportion of ingredients in the blend. This indicated a balance had to be struck to maximize fiber content while incorporating turnip powder. This study extended beyond mere nutritional analysis to discuss the implications of these blends for human health. It suggested that incorporating turnip powder could aid in reducing dietary fat intake and enriching fiber consumption, potentially mitigating risks associated with chronic diseases such as obesity and cardiovascular disorders. Further, recommendations were proposed for incorporating these blends into daily meals to enhance nutritional intake.

The decline in ash content, which reflected mineral content, suggested that the mineral contribution of turnips did not compensate for the reduction in wheat and oats, presumably richer in minerals. The marked decrease in NFE content from T_0_ to T_5_ indicated a carbohydrate reduction with increased turnip inclusion. Given that NFE represented the carbohydrate fraction, this suggested that turnips had a lower carbohydrate content than grains. This aligned with the works of Shewry and Hey (2015) on wheat's nutritional contributions, Warwick (2011) and Rasane et al. (2015) on the benefits of oats and turnips, and the emphasis on sustainable food production by Boye et al. (2010) and Remans et al. (2011).

The mineral analysis demonstrated significant variations across treatments, with calcium increasing and phosphorus, iron, and potassium decreasing mean values from T_0_ to T_5_. The increase in calcium content in T_5_ was particularly noteworthy, suggesting that turnips significantly contributed calcium (inherent calcium content) to the blend. Conversely, the decrease in phosphorus, iron, and potassium with higher turnip proportions indicated a dilution effect despite turnips being recognized as a good source of these nutrients.

The statistical analysis in this study showed highly significant differences (*p* < 0.01) for all measured parameters (moisture, protein, fat, fiber, ash, NFE, calcium, phosphorus, iron, and potassium) across the treatment groups. This indicated that varying the concentration of turnip powder in the wheat–oat blends significantly affected their proximate and mineral composition. The mean squares for proximate and mineral composition underscored the statistical significance of the observed changes, affirming the impact of turnip powder concentration on the nutritional profile of wheat–oat blends. The significant differences in moisture, protein, fat, fiber, ash, and NFE, alongside minerals like calcium, phosphorus, iron, and potassium, highlighted the need to consider the balance of ingredients to achieve desired nutritional outcomes. The paper demonstrated the potential of wheat–oat–turnip blends in offering a nutritious, sustainable, and consumer‐acceptable food product, contributing to the global pursuit of healthier eating habits and agricultural biodiversity.

The findings paved the way for future research into other vegetable and grain combinations, exploring broader implications for dietary practices and sustainability. Investigations into the sensory and nutritional impacts of varying concentrations of alternative vegetable powders within cereal‐based blends could offer new insights into the development of nutritionally balanced, sustainable food products. Additionally, research into consumer acceptance and practical applications of these blends in everyday meals would provide valuable data to inform dietary recommendations and product development.

## 5 Conclusions

The incorporation of turnip powder into wheat–oat blends demonstrated a promising approach to enhancing the nutritional profile of cereal‐based products. The results indicated that increasing the proportion of turnip powder significantly boosts calcium content, addressing dietary deficiencies while also introducing valuable vitamins and minerals. However, it was noted that the higher inclusion of turnip powder resulted in reductions of other nutritional components such as protein and fat due to the lower concentrations of these nutrients in turnips. Sensory evaluations revealed that a blend containing 30% turnip powder achieved the highest overall acceptability regarding taste, flavor, and texture. These findings underscored the potential for developing functional foods that not only meet consumer preferences but also contribute to healthier dietary practices. By effectively combining diverse crops, this study paved the way for further research into additional vegetable and grain blends, enriching dietary diversity and supporting agricultural sustainability. The integration of turnip powder stands as a viable strategy for addressing nutritional gaps while promoting wholesome, minimally processed food choices.

## Author Contributions

Itrat Fatima Toor: investigation, writing. Sanaullah Sajid: methodology, supervision. Amna Akmal: writing – review and editing. Zain Ul Abidin: validation. Zeenat Fatima and Tawfiq Alsulami: data curation. Suleiman Abdullah Althawab and Lianxian Guo: formal analysis, writing – review and editing.

## Conflicts of Interest

The authors declare no conflicts of interest.

## Data Availability

The corresponding author will share the data underlying this article at a reasonable request.
